# Role of CXCL13 and CCL20 in the recruitment of B cells to inflammatory foci in chronic arthritis

**DOI:** 10.1186/s13075-018-1611-2

**Published:** 2018-06-07

**Authors:** Estefanía Armas-González, María Jesús Domínguez-Luis, Ana Díaz-Martín, Mayte Arce-Franco, Javier Castro-Hernández, Gabriela Danelon, Vanesa Hernández-Hernández, Sagrario Bustabad-Reyes, Alberto Cantabrana, Mariagrazia Uguccioni, Federico Díaz-González

**Affiliations:** 1 Servicio de Reumatología, Hospital Universitario de Canarias, La Laguna, Tenerife Spain; 2 Institute for Research in Biomedicine, Università della Svizzera italiana, Bellinzona, Switzerland; 3 Servivio de Reumatología, Hospital Universitario de Canarias, La Laguna, Tenerife Spain; 4 Servicio de Reumatología, Hospital Universitario Nuestra Señora de Candelaria, Santa Cruz de Tenerife, Tenerife Spain; 5 Department of Biomedical Sciences, Humanitas University, Milan, Italy; 6 Departamento de Medicina Interna, Facultad de Medicina, Universidad de La Laguna, La Laguna, Tenerife Spain; 7 Servicio de Reumatología, Hospital Universitario de Canarias, C/Ofra s/n. La Cuesta, 38320 La Laguna, Tenerife Spain

**Keywords:** Rheumatoid arthritis, Psoriatic arthritis, B cells, Chemokines and chemokine receptors

## Abstract

**Background:**

B cells exert their pathogenic action in rheumatoid arthritis (RA) locally in the synovium. This study was undertaken to elucidate the chemokines responsible for the recruitment of B cells in the inflamed synovium, taking into account that the rich chemokine milieu present in the synovial tissue can fine-tune modulate discrete chemokine receptors.

**Methods:**

Expression levels of chemokine receptors from the CC and CXC family, as well as CD27, were assessed by flow cytometry in CD20^+^ mononuclear cells isolated from the peripheral blood (PB) and synovial fluid (SF) of RA and psoriatic arthritis patients. Transwell experiments were used to study migration of B cells in response to a chemokine or in the presence of multiple chemokines.

**Results:**

B cells from the SF of arthritis patients showed a significant increase in the surface expression of CCR1, CCR2, CCR4, CCR5 and CXCR4 with respect to PB. Conversely, SF B cells expressed consistently lower amounts of CXCR5, CXCR7 and CCR6, independent of CD27 expression. Analysis of permeabilized B cells suggested internalization of CXCR5 and CCR6 in SF B cells. In Transwell experiments, CCL20 and CXCL13, ligands of CCR6 and CXCR5, respectively, caused a significantly higher migration of B cells from PB than of those from SF of RA patients. Together, these two chemokines synergistically increased B-cell migration from PB, but not from SF.

**Conclusions:**

These results suggest that CXCL13 and CCL20 might play major roles in RA pathogenesis by acting singly on their selective receptors and synergistically in the accumulation of B cells within the inflamed synovium.

**Electronic supplementary material:**

The online version of this article (10.1186/s13075-018-1611-2) contains supplementary material, which is available to authorized users.

## Background

Rheumatoid arthritis (RA) and psoriatic arthritis (PsA) are systemic autoimmune diseases characterized by the chronic inflammation of joints which leads to the destruction of cartilage and bone [1]. Both T cells and B cells are present in the inflammatory infiltrate of synovial membranes in both diseases [1–3]. However, clinical and experimental evidence indicates that B cells play a dissimilar pathogenic role in RA and PsA; specifically, B lymphocytes are not believed to be a major factor in PsA pathophysiology, as they are in RA [4–6]. Experimental data suggest that B cells ought to migrate and accumulate in the synovial microenvironment in order to exert their pathogenic action in RA patients [7], through the local production of autoantibodies and proinflammatory soluble factors, as well as by acting as antigen-presenting cells [6, 8, 9]. However, the driving force responsible for the recruitment of B cells in the synovium of RA and PsA patients is not fully clarified. A better understanding of the mechanisms involved in the recruitment of B cells into inflamed synovial tissue might help identify potential therapeutic targets for the management of joint inflammatory processes such as RA.

A number of chemokines, low molecular weight cytokines, are produced locally in the target sites of leukocyte trafficking and homing [10, 11]. There are currently described more than 50 chemokines and 20 G-protein-coupled chemokine receptors [12]. Following the binding of a chemokine to its receptor, intracellular signal pathways lead to functional responses such as chemotaxis, secretion and transcriptional activation [12, 13]. Most leukocytes express more than one chemokine receptor of varying promiscuity and are, in addition, potentially capable of responding synergistically to concomitant migration signals [14]. Upon binding to their ligands, chemokine receptors are rapidly internalized following either a degradative or recycling pathway. This mechanism, known as desensitization [15], limits the magnitude and duration of signaling, thus protecting cells from chemokine overstimulation [16].

In arthritis, several chemokines and their receptors are involved in the process of leukocyte extravasation and accumulation in the inflamed synovium [17], as well as in the lymphoid tissue organization of synovial inflammatory infiltrate [18, 19]. Several chemokines have been identified in the synovial microenvironment of both RA [17, 20–22] and PsA patients [23–25]. While the individual effects of different chemokines are well known, the potential consequences of the concomitant expression of chemokines under pathologic conditions in vivo are still a matter of debate [26]. With respect to B cells, one study found that the chemokine system influences the development and inflammatory progression of B-cell-mediated autoimmune diseases [27], and that a rich chemokine environment strongly enhances in-vitro migration by an induced synergism involving this cell type [28].

The objective of this work was to determine which chemokines play the most relevant roles in the accumulation of B cells in the synovium of patients with RA and PsA. Based on CXCR5 and CCR6 internalization and B-cell migration experiments, our results suggest that CXCL13 and CCL20, the respective ligands of CXCR5 and CCR6, by acting synergistically, might participate in the recruitment of B cells in the synovium in patients with arthritis.

## Methods

### Patient and biological samples

Samples of peripheral blood (PB) and synovial fluid (SF) were obtained from 12 and 13 patients with clinically active RA and PsA, respectively (mean ± standard deviation 28-joint Disease Activity Score (DAS28) erythrocyte sedimentation rate 4.31 ± 0.68 and 4.70 ± 0.55, respectively). All patients met the American College of Rheumatology/European League Against Rheumatism classification criteria for RA [29] and the Classification Criteria for Psoriatic Arthritis (CASPAR) [30]. Patients attended our institution from December 2015 to June 2017, and were consecutively included in the study. Table 1 presents the clinical characteristics of the patients included in this study. PB and SF were simultaneously obtained by puncture of inflamed knees. All patients gave informed consent, and the study was approved by the Ethics Committee of Hospital Universitario de Canarias (Tenerife, Spain). The expression level of chemokine receptor in CD20^+^ PB mononuclear cells from seven healthy voluntaries was used as control.

**Table 1:** Clinical characteristics of patients

Patient	Sex	Age (years)	Diagnosis	Clinical pattern	RF	ACPA	Time of evolution (years)	Treatment	CC dose (mg/day)
1	F	46	RA	Polyarticular	Pos	Pos	11	Anti-TNF + MTX + PRED	5–10
2	F	55	RA	Polyarticular	Neg	Neg	9	MTX	0
3	F	77	RA	Polyarticular	Pos	Pos	8	MTX + OH CLQ + DEFL	6
4	F	51	RA	Polyarticular	Neg	Neg	8	MTX	0
5	M	33	RA	Polyarticular	Neg	ND	15	Anti-TNF + MTX	0
6	M	25	RA	Polyarticular	Neg	Neg	0	None	0
7	M	17	RA	Polyarticular	Neg	Neg	14	Anti-TNF + MTX	0
8	F	53	RA	Polyarticular	Pos	Pos	16	MTX + PRED	7.5
9	F	62	RA	Polyarticular	Pos	Pos	7	Anti-TNF + MTX + PRED	5
10	M	70	RA	Polyarticular	Neg	ND	7	PRED	5
11	F	98	RA	Polyarticular	Pos	Pos	11	Anti-IL-6 + PRED	5
12	M	71	RA	Polyarticular	Pos	Pos	8	PRED	5
13	F	47	PsA	Polyarticular	Neg	ND	16	MTX + NSAID	0
14	M	52	PsA	Polyarticular	Neg	ND	12	MTX + DEFL	7.5
15	M	53	PsA	Polyarticular	Neg	Neg	13	MTX + DEFL	15
16	M	53	PsA	Polyarticular	Neg	Neg	13	MTX + DEFL	15
17	M	58	PsA	Oligoarticular	Neg	Neg	0	None	0
18	F	39	PsA	Polyarticular	Neg	ND	5	Anti-IL-17	0
19	F	31	PsA	Polyarticular	Neg	ND	7	PRED	5
20	M	46	PsA	Oligoarticular	Neg	Neg	6	PRED	7.5
21	F	32	PsA	Polyarticular	Neg	ND	8	PRED	5
22	M	25	PsA	Oligoarticular	Neg	Neg	0	None	0
23	M	55	PsA	Oligoarticular	Neg	ND	6	Anti-TNF + MTX	0
24	M	54	PsA	Polyarticular	Neg	Neg	7	Anti-TNF + MTX	0
25	F	65	PsA	Oligoarticular	Neg	Neg	40	NSAID	0

*ACPA* anti-citrullinated peptide antibodies, *CC* corticosteroid, *DEFL* deflazacort, *F* female, *IL* interleukin, *M* male, *MTX* methotrexate, *ND* not determined, *Neg* negative, *NSAID* nonsteroidal antiinflammatory drug, *PsA* psoriatic arthritis, *Pos* positive, *PRED* prednisone, *OH CLQ* hydroxycloroquine, *RA* rheumatoid arthritis, *RF* rheumatoid factor, *TNF* tumor necrosis factor

B cells from healthy donors were isolated by immunoselection (see later) using buffy coats provided by the Instituto de Hemodonación y Hemoterapia (Tenerife, Spain).

### Cell isolation and culture

Mononuclear cells were isolated from heparinized PB and SF samples by Biocoll (Biochrom AG, Berlin, Germany) density-gradient centrifugation (300 × *g*, at room temperature for 30 min). After washing in phosphate buffer without calcium and magnesium (PBS), B cells were selected by anti-human CD20 or anti-human CD19 fluorescein monoclonal antibodies for flow cytometry analysis. For specific experiments, B cells were isolated from the buffy coats of healthy donors by negative (StemCell Technologies Inc., Vancouver, Canada) or positive (Miltenyi Biotec, Bergisch Gladbach, Germany) immunoselection techniques, as indicated, using magnetic beads, resulting in a purity > 95%, which was ascertained by positivity to CD19 or CD20 using flow cytometric analysis.

### Flow cytometry analysis

#### Surface labeling of chemokine receptors

Mononuclear cells freshly isolated from PB and SF were simultaneously labeled with two directly conjugated mAbs at 4 °C for 30 min. One of these was fluorescein isothiocyanate (FICT)-conjugated anti-human CD20mAb (2H7 clone; BD Pharmigen, Franklin Lakes, NJ, USA), which was used to select the B-cell subset; and the other was either a phycoerythrin (PE)-conjugated anti-human mAb against CCR1 (53504 clone; R&D Systems, Minneapolis, MN, USA), CCR2 (48607 clone; R&D), CCR3 (61828 clone; R&D), CCR4 (20540 clone; R&D), CCR5 (2D7 clone; BD Pharmigen) and CXCR4 (12G5 clone; BD Pharmigen), or an allophycocyanin (APC)-conjugate anti-human mAb against CCR6 (53101 clone; R&D), CCR7 (150503 clone; R&D), CXCR2 (6C6 clone; BD Pharmigen), CXCR5 (51505 clone; R&D), CXCR6 (56811 clone; R&D) and CXCR7 (358426 clone; R&D).

In selective experiments, mononuclear PB and SF cells were simultaneously incubated with three monoclonal antibodies directly conjugated to different fluorochromes for 30 min at 4 °C. An anti-human CD20 FITC mAb (2H7 clone; BD Pharmigen) was used to select the B-cell population combined with an anti-human CD27 PE mAb (M-T271 clone; BD Pharmigen), to distinguish between memory (CD27^+^) and naïve (CD27^–^) B cells. Cells were also simultaneously stained with human mAb against CXCR4 APC (12G5 clone; BD Pharmigen), CXCR5 APC (51505 clone; R&D), CXCR7 APC (11G8 clone; R&D) or CCR6 APC (REA190 clone; Miltenyi Biotec). The fluorescence levels of isotype-matching antibodies (ImmunoTools GmbH, Friesoythe, Germany) were used as controls. After washing with PBS, at least 2 × 10^4^ lymphocytes from each sample were analyzed using an Accuri C6 flow cytometer (BD Biosciences) and data were analyzed using BD Accuri C6 software.

Data of chemokine expression were presented as the percentage of positive cells and mean fluorescence intensity (MFI), using the fluorescence of isotype-matching antibody as controls. Because fluorescence conditions varied between experiments, the data were normalized to express the relative mean fluorescence intensity (rMFI) according to the equation:

$$ \mathrm{rMFI}=\left(\left({\mathrm{MFI}}_{\mathrm{SF}}\hbox{--} {\mathrm{MFI}}_{\mathrm{isotype}\ \mathrm{control}}\right)/\left({\mathrm{MFI}}_{\mathrm{PB}}\hbox{--} {\mathrm{MFI}}_{\mathrm{isotype}\ \mathrm{control}}\right)\right)\times 100. $$

#### Extracellular and intracellular labeling of chemokine receptors

In selective experiments, the expression of CXCR4, CXCR5 and CCR6 on CD20^+^ cells from PB and SF was assessed in both nonpermeabilized (surface expression) and permeabilized (total expression: surface plus intracellular) lymphocytes using double labeling, as described previously. Mononuclear cells were freshly and rapidly isolated after extraction of PB and SF and were maintained at 4 °C to avoid receptor internalization [31]. Cells were permeabilized using a Cytofix/Cytoperm permeabilization/fixation kit (BD Biosciences). After washing, 3 × 10^4^ lymphocytes from each sample were analyzed using the Accuri C6 cytometer (BD Biosciences). Data were normalized and expressed as rMFI, being the surface or total expression of each chemokine receptor on B cells from PB considered 100%.

### Migration assays

#### Transwell experiments

The migration capacity of B-cells was studied using a Transwell 96-well permeable support system with a polycarbonate membrane with pores 3 μm in diameter (Corning, NY, USA). CD19^+^ B cells were isolated by a negative immunoselection process, using the EasySep™ Human CD19 Selection Kit (Stem Cell). Chemoattractants and freshly isolated B cells (1.5 × 10^5^ cells/well), both in RPMI 1640 supplemented with 20 mM HEPES, pH 7.4, and 0.5% bovine serum albumin (BSA), were placed into the lower and upper wells, respectively. To identify the optimum migration concentration, dose–response assays were performed for the recombinant human chemokines CXCL13 (R&D Systems) and CCL20 (ImmunoTools, GmbH), using 10, 100 and 1000 nM. Then, increasing concentrations of CXCL13 were used in the presence of the optimum concentration of CCL20 and vice versa. As a control, the anti-human CXCR5 blocking antibody (51505 clone; R&D) was used at a concentration of 1.5 μg/ml in the presence of 1000 nM CXCL13 alone, or in a mixture of 1000 nM CXCL13 plus 1000 nM CCL20. After 3 h of incubation at 37 °C, the content of the lower compartment was collected and the number of migrated cells was analyzed in 50 μl of each sample using a medium acquisition speed in the Accuri C6 cytometer.

The migration capacity of B cells from PB and SF of arthritis patients was also studied using a Transwell six-well system with pores 3 μm in diameter (Corning). CXCL13, CCL20, CXCL12 and a mixture of CXCL13 and CCL20, all at 1000 nM, and freshly isolated mononuclear cells (5 × 10^6^ cells/well), both in medium as described, were placed into the lower and upper wells, respectively. After 3 h of incubation at 37 °C, the cells of the lower compartment were labeled with anti-human CD20 FITC for 30 min at 4 °C, washed in PBS and analyzed by flow cytometry. B-cell migration to the lower well was assessed with respect to the total number of B cells that were initially loaded into the upper well, identified by anti-CD20 FITC labeling of freshly isolated mononuclear cells from PB or SF.

### Statistical analysis

Differences between groups were analyzed for statistical significance using the *t* test for paired (differences between PB and SF in patients) or unpaired (differences between patients and controls) samples. *p* < 0.05 was considered significant. Results are expressed as the arithmetic mean ± standard error (SE) of the mean.

## Results

### Chemokine receptors are differentially expressed on B cells from PB and SF of RA and PsA patients

**Fig. 1:**
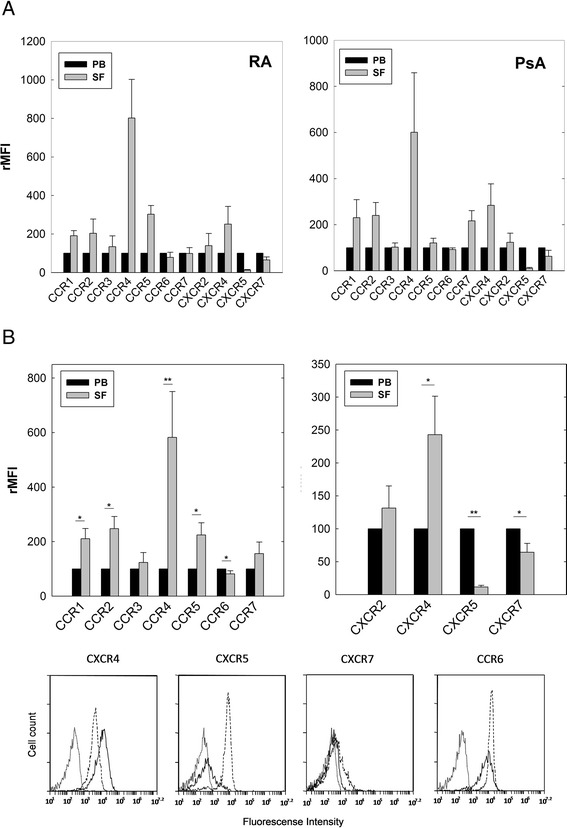
Surface expression of chemokine receptors in CD20^+^ cells from PB and SF of RA and PsA patients. Mononuclear cells isolated from PB and SF labeled with two fluorochrome-conjugated mAbs and analyzed by flow cytometry. **a** Surface expression of chemokine receptors in CD20^+^ B cells from PB and SF of patients with RA (left) and PsA (right). Data represent mean ± SE of rMFI from six and five independent experiments, respectively. Expression level of each chemokine receptor in PB B cells considered 100%. **b** Surface expression of chemokine receptors in CD20^+^ B cells from PB and SF of patients with RA and PsA plotted together. Data represent mean ± SE of rMFI from 11 independent experiments. Representative flow cytometry histograms showing surface expression of CXCR4, CXCR5, CXCR7 and CCR6 in CD20^+^ cells from PB and SF of patients with RA. Dotted histograms represent isotype-matching control; dashed and closed histograms represent expression in B cells from PB and SF, respectively. *p < 0.05, **p < 0.01 by *t* test for paired samples. PB peripheral blood, PsA psoriatic arthritis, RA rheumatoid arthritis, SF synovial fluid, rMFI relative mean fluorescence intensity

Chemokines and their receptors constitute a chemotactic system that controls the migratory patterns and positioning of all immune cells [12]. To investigate the role of this system in the migration of B cells to the inflamed synovial tissue, we first studied by flow cytometry the surface expression of a number of chemokine receptors from the CC and CXC family on CD20^+^ mononuclear cells isolated simultaneously from PB and SF of patients with active RA and PsA. When the surface expression level data expressed as rMFI were plotted, either by disease diagnosis (Fig. 1a) or grouped (Fig. 1b), significant increments of CCR1, CCR2, CCR4, CCR5 and CXCR4 were observed on CD20^+^ cells from SF with respect to PB. Conversely, a significant and consistent decrease in the surface expression of CXCR5, CXCR7 and CCR6 was detected in the B cells from SF compared to PB, independent of whether the analysis examined the diseases separately or together (Fig. 1a, b). The expression profile of chemokine receptors on B cells from PB and SF of RA patients was independent of RF presence or absence (data not shown). Data of Fig. 1b are also displayed in Additional file 1: Table S1 as the raw MFI and percentage of positive cells. The chemokine expression on PB B cells, as assessed by MFI or proportion of positive cells, was not significantly different between arthritic patients and healthy controls, except for CCR6 which was significantly higher expressed in B cells from patients than from controls.

These data demonstrate that B cells recruited in inflamed joints of RA and PsA patients modify in a similar manner their basal surface expression profile of chemokine receptors.

### Synovial B cells increase CXCR4 and decrease CXCR5, CCR6 and CXCR7 surface expression, independent of their naïve or memory phenotype

**Fig. 2:**
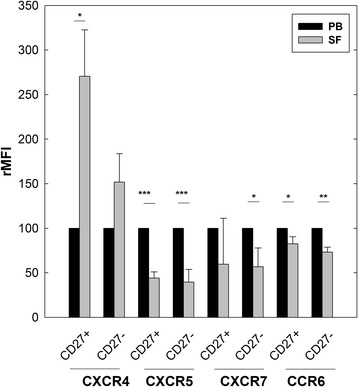
Surface expression of CXCR4, CXCR5, CXCR7 and CCR6 on memory (CD27^+^) and naïve (CD27^–^) B cells from PB and SF of patients with arthritis. Mononuclear cells isolated from PB and SF labeled with a combination of three fluorochrome-conjugated mAbs and analyzed by flow cytometry. Surface expression of chemokine receptors studied in CD27^+^ and CD27^–^ in CD20^+^ cells from PB (considered 100%) and SF in patients with RA. Data expressed as mean ± SE of rMFI from seven independent experiments compared to PB expression of each chemokine receptor, considered 100%. *p < 0.05, **p < 0.01, ***p < 0.001 by *t* test for paired samples. rMFI relative mean fluorescence intensity, PB peripheral blood, SF synovial fluid

The expression levels of several chemokine receptors are regulated during cell differentiation and maturation [32]. Therefore, we studied the expression of CXCR4 (an upregulated receptor) and CXCR5, CXCR7 and CCR6 (three downregulated receptors in SF B cells) on CD20^+^ cells from PB and SF depending on whether they had been in contact (CD27^+^) or not (CD27^–^) with the antigen [33]. Flow cytometry analysis showed a higher percentage of memory (CD27^+^) versus naïve (CD27^–^) B cells in SF (CD27^+^ 73 ± 3.66% versus CD27^–^ 29 ± 3.21%, *p* < 0.01) compared to PB (CD27^+^ 31 ± 3.89 versus CD27^–^ 70 ± 3.91, *p* < 0.01) with no differences found between RA and PsA patients. Triple-color flow cytometric analysis showed that, in general, there were no significant differences in the expression of these chemokine receptors between CD27^+^ and CD27^–^ B lymphocytes from PB and SF of RA patients (Fig. 2). Expression of CXCR4 showed a significant increase in CD27^+^ and CD27^–^ cells from SF with respect to PB, while there was a consistent decrease in the expression of CXCR5, CCR6 and CXCR7 in SF B cells with respect to PB, independent of their CD27 phenotype (Fig. 2). Table 2 presents the differences in the absolute MFI using flow cytometric analysis of CXCR4, CXCR5, CXCR7 and CCR6 on CD27^+^ and CD27^–^ B cells between PB and SF from RA patients. Similar results were obtained in PsA patients (data not shown).

**Table 2:** Chemokine receptor expression on memory (CD27^+^) and naïve (CD27^–^) CD20^+^ cells from SF and PB of patients with rheumatoid arthritis

	CXCR4			CXCR5			CXCR7			CCR6		
	CD27^+^	CD27^–^		CD27^+^	CD27^–^		CD27^+^	CD27^–^		CD27^+^	CD27^–^	
PB	1746 ± 177	3648 ± 247	***p*** **= 0.001**	12,870 ± 719	16,028 ± 1102	***p*** **= 0.003**	232 ± 36	349 ± 86	*p* = 0.15	10,243 ± 1143	10,130 ± 1792	*p* = 0.94
SF	4821 ± 1250	5880 ± 1531	*p* = 0.09	5568 ± 861	6239 ± 701	*p* = 0.26	105 ± 91	93 ± 25	*p* = 0.9	8040 ± 1041	6932 ± 1487	*p* = 0.37
	***p*** **= 0.028**	*p* = 0.14		***p*** **= 0.0001**	***p*** **= 0.0001**		*p* = 0.33	***p*** **= 0.04**		***p*** **= 0.02**	***p*** **= 0.005**	

Values are mean ± standard error of mean fluorescence intensity of each chemokine receptor on memory and naïve B cells from PB and SF. Bold data statistically significant at *p* < 0.05

*PB* peripheral blood, *SF* synovial fluid

These data show that expression profiles of the chemokine receptors CXCR4, CXCR5, CXCR7 and CCR6 in synovial B cells, compared to those of PB, were not modified by previous contact with the antigen.

### Synovial B cells from RA patients internalize CXCR5 and CXCR6 receptors

**Fig. 3:**
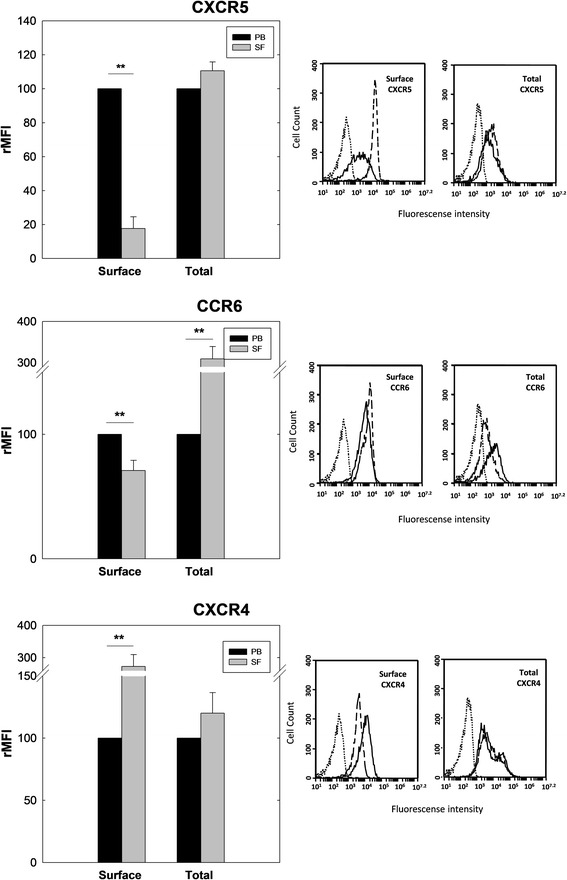
Surface and total expression of CXCR5, CCR6 and CXCR4 in B lymphocytes from PB and SF of patients with RA. Mononuclear cells isolated from PB and SF labeled with a combination of two fluorochrome-conjugated mAbs and analyzed by flow cytometry. Surface (nonpermeabilized) and total (permeabilized) expression of chemokine receptors studied in CD20^+^ cells from PB and SF of RA patients. Data expressed as mean ± SE of rMFI from five independent experiments compared to PB expression of each chemokine receptor, considered 100%. Representative flow cytometry histograms showing surface expression of CXCR5, CCR6 and CXCR4 in CD20^+^ cells from PB and SF of a patient with RA. Dotted histograms represent isotype-matching control; dashed and closed histograms represent expression in B cells from PB and SF, respectively. **p < 0.01, *t* test for paired samples. rMFI relative mean fluorescence intensity, PB peripheral blood, SF synovial fluid

It is well established that the recognition of ligand by chemokine receptors causes a decrease in their surface expression due to receptor internalization [16]. B lymphocytes present in the SF of patients with active arthritis showed a significant reduction of CXCR5 and CCR6 receptors. To determine whether this reduction was due to an internalization mechanism, we used flow cytometry to study the expression of both receptors in nonpermeabilized and permeabilized CD20^+^ cells from PB and SF of RA patients. Our results showed that the differences observed in CXCR5 and CCR6 on nonpermeabilized cells (surface expression) between B cells from PB and SF tended to disappear, or even become inverted, when their expression was assessed in permeabilized cells (total expression) (Fig. 3). This relationship, when measured as a percentage of the mean fluorescence intensities in nonpermeabilized CD20^+^ cells, showed that CXCR5 and CCR6 surface expression levels were 33 ± 5% and 76 ± 5% in SF with respect to PB (considered 100%), respectively. However, in permeabilized B cells the total expression of CXCR5 was equalized between SF (108 ± 5%) and PB, although total expression of CCR6 in SF increased above that of PB reaching 308 ± 35%. We also analyzed the surface and total expression of CXCR4, a chemokine receptor that increases its expression on B cells from the synovial microenvironment. Surface expression of CXCR4 reached 180 ± 16% on SF B cells with respect to that of PB, although in permeabilized B cells this difference in the expression levels between the two compartments was not observed.

These data suggest that CXCR5 and CCR6, but not CXCR4, reduce their surface expression on synovial B cells by internalization, a process that can be interpreted in the context of desensitization to CXCL13 and CCL20, but not to CXCL12.

### CXCL13 and CCL20 exert a synergistic effect on B-lymphocyte migration

A synergistic effect has been described among several chemokines in vitro and in vivo [34] resulting in increased leukocyte migration [14]. Therefore, we studied the chemoattractant capacity of CXCL13 and CCL20 on B cells using transmigration assays.

**Fig. 4:**
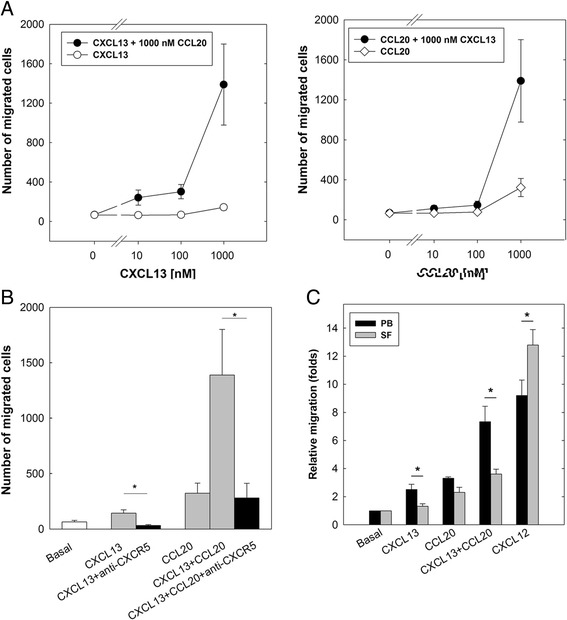
CXCL13 and CCL20 exhibit synergistic effect on transmigration of B lymphocytes in vitro. **a** Migration in Transwell experiments of CD19^+^ B cells from PB of healthy donors. Chemotaxis induced by presence of CXCL13 (open circles) and CCL20 (open diamonds), as well as by their combination at different concentrations (filled circles). **b** Migration in Transwell experiments of CD19^+^ cells in presence of 1000 nM of CXCL13 or a combination of 1000 nM of CXCL13 and CCL20 in presence (black bars) or absence (gray bars) of blocking anti-CXCR5mAb. **a, b** Data represent absolute number of migrated cells obtained in triplicate and expressed as mean ± SE of four independent experiments. **c** Migration of CD20^+^ cells from PB and SF of RA patients. Data represent relative number of migrated B cells from PB and SF in Transwell experiments compared to basal conditions, considered 1. Data expressed as mean ± SE of five independent experiments. *p < 0.05, by *t* test for paired samples. PB peripheral blood, SF synovial fluid

B cells isolated from buffy coats of healthy donors were used for Transwell experiments. The optimal cell migration was obtained at 1000 nM CXCL13 and 1000 nM CCL20. When B cells were incubated at the optimum concentration for CCL20 or CXCL13 (1000 nM) in combination with increasing concentrations of the other chemokine (10, 100 or 1000 nM), a clearly synergistic effect was observed in B-cell migration (five-fold increase), in particular when a mixture containing 1000 nM of both chemokines was used as a chemoattractant (Fig. 4a). The presence of a blocking anti-CXCR5 monoclonal antibody in Transwell experiments abrogated the transmigration effect of CXCL13 on B cells, either when it was used alone or in combination with CCL20 (Fig. 4b).

### Synovial B cells from RA patients show a decreased migration capability in response to CXCL13 and CCL20 compared to that from PB

The aforementioned results demonstrated that CXCL13 and CCL20 act synergistically in the migration of PB B cells from healthy donors. To determine whether B cells from PB and SF of RA patients behave like those from PB of healthy donors, mononuclear cells from patients with RA were isolated and incubated in Transwells in the presence of an optimal concentration (1000 nM) of CXCL13 and CCL20 and with both chemokines in combination. Fig. 4c shows that B cells from PB exert a higher migration capability in the presence of CXCL13 and CCL20 than B cells from SF. The combination of both chemokines showed a synergistic effect in PB from RA patients, similar to that observed in healthy controls, but not in SF B cells. Conversely, migration of B cells in response to CXCL12 a ligand of CXCR4, was higher in B cells from SF than from PB of RA patients.

These results suggest that CXCL13 and CCL20, acting synergistically, are the main responsible driving force in the migration and accumulation of B cells in the inflamed synovium of RA patients.

## Discussion

The most important findings of this work can be summarized as follows. B cells present in the synovial microenvironment of patients with RA and PsA express the chemokine receptors CXCR5, CCR6 and CXCR7, albeit in significantly lower amounts than those in PB, independent of disease type and their naïve or memory phenotype. Also, this reduction in surface expression is caused by internalization and not due to other forms of downmodulation. Finally, CXCL13 and CCL20, the respective ligands of CXCR5 and CCR6, show a synergistic effect in driving the migration of PB B cells from healthy donors and RA patients. Taken together, these results strongly suggest that CXCL13 and CCL20, acting synergistically, play a key role in the recruitment of B cells in the inflamed synovium.

It has been reported that the accumulation of B cells in the synovium plays an essential role in the pathogenesis of RA [7]. However, evidence suggests that this cell type does not seem to play a major role in PsA pathogenesis [4–6]. Once in the rheumatoid joint, B cells participate in the pathogenesis of the disease through different mechanisms [8, 9]. Although a wide variety of chemokines and their receptors are expressed in the synovium of RA [17, 20, 21] and PsA patients [23–25], which of the chemokines present in the synovial microenvironment play a predominant role in the accumulation of B lymphocytes in the joints of these patients remains to be fully clarified.

In order to investigate which chemokines play a major role in the accumulation of B cells in the inflamed synovium, we first studied the surface expression profile of different chemokine receptors from the CC and CXC family in B cells isolated from PB and SF. Our data showed that the expression of chemokine receptors on B cells present in the inflamed synovia is differentially modulated, either positively or negatively, both in RA and PsA patients. B cells from SF showed significant upregulation of CCR1, CCR2, CCR4, CCR5 and CXCR4, as well as downregulation of CXCR5, CXCR7 and CCR6 surface expression compared to PB B cells in both diseases. Different chemokine receptors have been described on PB B cells from healthy individuals and patients with autoimmune diseases, such as RA and systemic lupus erythematosus (SLE) [35]. However, only two studies have compared chemokine expression on B cells from PB and SF, one in RA patients [36] and the other in juvenile idiopathic arthritis (JIA) patients [37]. As with our own findings, the synovial B cells in these two studies less often expressed CXCR5 and CCR6 compared to PB. Our data for the proportion of B cells positive for CCR5, CCR6, CXCR4 and CXCR5 in both PB and the synovial microenviroment from arthritic patients and healthy controls are compatible with those described previously by Nanki et al. [36]. However, although the same mAb was used, Nanki et al.’s work show that more than 60% of B cells from patients and controls express CCR7, and ours show that this chemokine receptor is much less expressed on B cells from both patients and controls. As far as we know, a comparative analysis of chemokine expression between B cells in PB and SF from PsA patients has not been studied previously. In addition, this marks the first time that the surface expression levels of CCR1, CCR3, CCR4 and CXCR7 have been analyzed in SF and PB B cells from RA patients.

The expression of certain chemokine receptors is regulated during cell differentiation and maturation [32, 38]. When B lymphocytes from RA patients were selected based on their memory (CD27^+^) or naïve (CD27^–^) phenotype, SF B cells expressed consistently lower levels of CXCR5, CXCR7 and CCR6 and higher CXCR4 levels compared to PB regardless of their prior contact with the antigen. The study by Nanki et al. [36] analyzing the percentage of positive cells, but not MFI, described similar results, except for CXCR4 which was expressed by 100% of B cells from both compartments, as was the case in our own study.

Since chemokine receptors rapidly reduce their surface expression upon ligand binding [16], it is reasonable to think that the chemokine that causes the downregulation of its receptor in the inflammatory foci should play a relevant biological role; for instance and in our context, in the recruitment of B cells to the joints. The surface expression of CXCR5, CXCR7 and CCR6 were decreased in SF B cells compared to PB. This finding is consistent with a desensitization phenomenon, as a consequence of receptor occupancy by their respective ligands [16]: CXCL13, CXCL12 and CCL20, chemokines that are present in the synovial microenvironment [17, 20, 21]. Interestingly, the remaining receptors studied (CCR1, CCR2, CCR4, CCR5 and CXCR4) showed an increased surface expression on SF B cells compared to PB, although many of their respective ligands were also present in the SF [17]. The explanation for this is elusive since multiple regulatory mechanisms are involved in chemokine receptor signaling and expression [39, 40]. When we examined the differential expression of CXCR5, CCR6 and CXCR4 on the cell surface and total expression on B cells from the PB and SF of RA patients, we observed that CXCR5 and CCR6 in SF B cells had undergone an internalization process, whereas CXCR4 had not. We interpreted these results as follows: the receptors CXCR5 and CCR6 play a predominant role in the migration of naïve B cells and memory B cells to the inflamed synovium.

CXCL12 is a chemokine abundantly expressed in the synovial tissue of RA patients [17]. Based on migration experiments using PB B cells from healthy donors, it has been proposed that CXCL12 might play a relevant role in B-cell migration into the rheumatoid synovium [19, 36]. However, our results tended toward a different direction; CXCR4 is strongly upregulated on the cell surface of SF B cells, a finding counter to the signaling by CXCL12 in these cells. Furthermore, our data on CXCR7 regulation in SF B cells go in the same direction. CXCR7 is an “atypical” chemokine receptor that acts as a decoy receptor for CXCL12 and CXCL11. The expression level of CXCR7 was significantly lower in SF B cells than in PB, suggesting that in the synovial microenvironment this B-cell receptor sequesters CXCL12, consequently reducing the availability of this chemokine for binding with CXCR4.

CXCL13 and CCL20, specific ligands of CXCR5 and CCR6, respectively, have been detected in the synovium of patients with chronic arthritis [17, 21, 41]. CXCL13 has been described as the most effective chemoattractant for B cells [42] and CCL20 as a selective chemoattractant factor for lymphocytes [43] expressing CCR6 receptors, such as naïve B cells [44]. Migration experiments showed that these two chemokines are chemoattractant for PB B cells from healthy donors. Synergistic effects among several chemokines have been described in vitro, which translates into increased leukocyte migration [14]. In this study, we present evidence of a novel positive regulatory mechanism of leukocyte migration in which the concomitant presence of adequate concentrations of CXCL13 and CCL20 synergistically increases the chemotaxis of B cells in vitro. We postulate that this type of synergy is mediated by dual receptors in which CXCR5 and CCR6 become activated following simultaneous or sequential binding of their agonists [14]. However, this issue should be confirmed by additional experimentation. Concurrently with these results, in Transwell experiments B cells from the PB of RA patients migrated to a significantly greater degree in the presence of CXCL13 and CCL20 than at baseline, and synergistically in the presence of both chemoattractants. However, the ability of B cells from SF of these patients to migrate in the presence of these chemokines was significantly lower than that of PB, which suggests that on B cells CXCR5 and CCR6 undergo desensitization in the synovial microenvironment. Conversely, B cells from SF showed very high migration ability in response to CXCL12, even higher than those from PB. This is an interesting finding that suggests additional regulatory mechanisms for CXCR4 expression and function, occurring in B cells migrated to the inflamed synovial tissue. Although the data obtained in both expression of receptors and migration are very consistent among patients, a limitation of this study is that the sample size does not allow the analysis of patients according to the use of steroids or DMARDs.

## Conclusions

Taken together, these results support the contention that CXCL13 and CCL20, acting synergistically, may constitute a key driving force for the migration and recruitment of B lymphocytes in the inflamed synovium of patients with arthritis. Given the relevant role that B cells have shown in the pathogenesis of RA [9, 45], the simultaneous interference of these two chemokines may represent a novel therapeutic target for the management of RA through the blockade of B-cell recruitment in the rheumatoid synovium.

## Additional file

Additional file 1: **Table S1.** Mean fluorescence intensity and percentage of positive cells for each chemokine receptor on PB and SF from arthritis patients and healthy controls. (DOCX 16 kb)

## Abbreviations

CASPAR: Classification Criteria for Psoriatic Arthritis
DAS28: 28-joint Disease Activity Score
DEFL: Deflazacort
DMARD: Disease-modifying antirheumatic drug
JIA: Juvenile idiopathic arthritis
mAb: Monoclonal antibody
MFI: Mean fluorescence intensity
MTX: Methotrexate
PB: Peripheral blood
PBS: Phosphate buffered saline
PsA: Psoriatic arthritis
RA: Rheumatoid arthritis
RF: Rheumatoid factor
rMFI: Relative mean fluorescence intensity
SE: Standard error
SF: Synovial fluid
SLE: Systemic lupus erythematosus

## References

[1] Smolen JS, Aletaha D, McInnes IB (2016). Rheumatoid arthritis. Lancet.

[2] Fitzgerald O, Winchester R (2009). Psoriatic arthritis: from pathogenesis to therapy. Arthritis Res Ther..

[3] Celis R, Planell N, Fernandez-Sueiro JL, Sanmarti R, Ramirez J, Gonzalez-Alvaro I (2012). Synovial cytokine expression in psoriatic arthritis and associations with lymphoid neogenesis and clinical features. Arthritis Res Ther..

[4] Edwards JC, Szczepanski L, Szechinski J, Filipowicz-Sosnowska A, Emery P, Close DR (2004). Efficacy of B-cell-targeted therapy with rituximab in patients with rheumatoid arthritis. N Engl J Med.

[5] Jimenez-Boj E, Stamm TA, Sadlonova M, Rovensky J, Raffayova H, Leeb B (2012). Rituximab in psoriatic arthritis: an exploratory evaluation. Ann Rheum Dis.

[6] Armas-Gonzalez E, Diaz-Martin A, Dominguez-Luis MJ, Arce-Franco MT, Herrera-Garcia A, Hernandez-Hernandez MV (2015). Differential antigen-presenting B cell phenotypes from synovial microenvironment of patients with rheumatoid and psoriatic arthritis. J Rheumatol.

[7] Takemura S, Klimiuk PA, Braun A, Goronzy JJ, Weyand CM (2001). T cell activation in rheumatoid synovium is B cell dependent. J Immunol.

[8] Moura RA, Graca L, Fonseca JE (2012). To B or not to B the conductor of rheumatoid arthritis orchestra. Clin Rev Allergy Immunol.

[9] Finnegan A, Ashaye S, Hamel KM (2012). B effector cells in rheumatoid arthritis and experimental arthritis. Autoimmunity.

[10] Luster AD (1998). Chemokines—chemotactic cytokines that mediate inflammation. N Engl J Med.

[11] Gerard C, Rollins BJ (2001). Chemokines and disease. Nat Immunol.

[12] Griffith JW, Sokol CL, Luster AD (2014). Chemokines and chemokine receptors: positioning cells for host defense and immunity. Annu Rev Immunol.

[13] Thelen M (2001). Dancing to the tune of chemokines. Nat Immunol.

[14] Proudfoot AE, Uguccioni M (2016). Modulation of chemokine responses: synergy and cooperativity. Front Immunol.

[15] Bennett LD, Fox JM, Signoret N (2011). Mechanisms regulating chemokine receptor activity. Immunology.

[16] Marchese A (2014). Endocytic trafficking of chemokine receptors. Curr Opin Cell Biol.

[17] Szekanecz Z, Koch AE, Tak PP (2011). Chemokine and chemokine receptor blockade in arthritis, a prototype of immune-mediated inflammatory diseases. Neth J Med.

[18] Manzo A, Paoletti S, Carulli M, Blades MC, Barone F, Yanni G (2005). Systematic microanatomical analysis of CXCL13 and CCL21 in situ production and progressive lymphoid organization in rheumatoid synovitis. Eur J Immunol.

[19] Corsiero E, Bombardieri M, Manzo A, Bugatti S, Uguccioni M, Pitzalis C (2012). Role of lymphoid chemokines in the development of functional ectopic lymphoid structures in rheumatic autoimmune diseases. Immunol Lett.

[20] Iwamoto T, Okamoto H, Toyama Y, Momohara S (2008). Molecular aspects of rheumatoid arthritis: chemokines in the joints of patients. FEBS J.

[21] Endres M, Andreas K, Kalwitz G, Freymann U, Neumann K, Ringe J (2010). Chemokine profile of synovial fluid from normal, osteoarthritis and rheumatoid arthritis patients: CCL25, CXCL10 and XCL1 recruit human subchondral mesenchymal progenitor cells. Osteoarthr Cartil.

[22] Manzo A, Vitolo B, Humby F, Caporali R, Jarrossay D, Dell'accio F (2008). Mature antigen-experienced T helper cells synthesize and secrete the B cell chemoattractant CXCL13 in the inflammatory environment of the rheumatoid joint. Arthritis Rheum.

[23] Muntyanu A, Abji F, Liang K, Pollock RA, Chandran V, Gladman DD (2016). Differential gene and protein expression of chemokines and cytokines in synovial fluid of patients with arthritis. Arthritis Res Ther..

[24] Devito A (2014). Interferon γ-induced chemokines in psoriatic arthritis. Clin Ter.

[25] Valcamonica E, Chighizola CB, Comi D, De Lucia O, Pisoni L, Murgo A (2014). Levels of chemerin and interleukin 8 in the synovial fluid of patients with inflammatory arthritides and osteoarthritis. Clin Exp Rheumatol.

[26] Cecchinato V, D'Agostino G, Raeli L, Uguccioni M (2016). Chemokine interaction with synergy-inducing molecules: fine tuning modulation of cell trafficking. J Leukoc Biol.

[27] Mitchison NA, Wedderburn LR (2000). B cells in autoimmunity. Proc Natl Acad Sci U S A.

[28] Paoletti S, Petkovic V, Sebastiani S, Danelon MG, Uguccioni M, Gerber BO (2005). A rich chemokine environment strongly enhances leukocyte migration and activities. Blood.

[29] Aletaha D, Neogi T, Silman AJ, Funovits J, Felson DT, Bingham CO (2010). 2010 Rheumatoid arthritis classification criteria: an American College of Rheumatology/European League Against Rheumatism collaborative initiative. Arthritis Rheum.

[30] Taylor W, Gladman D, Helliwell P, Marchesoni A, Mease P, Mielants H (2006). Classification criteria for psoriatic arthritis: development of new criteria from a large international study. Arthritis Rheum.

[31] Anselmo A, Mazzon C, Borroni EM, Bonecchi R, Graham GJ, Locati M (2014). Flow cytometry applications for the analysis of chemokine receptor expression and function. Cytometry A.

[32] Krzysiek R, Lefevre EA, Bernard J, Foussat A, Galanaud P, Louache F (2000). Regulation of CCR6 chemokine receptor expression and responsiveness to macrophage inflammatory protein-3alpha/CCL20 in human B cells. Blood.

[33] Klein U, Rajewsky K, Kuppers R (1998). Human immunoglobulin (Ig)M+IgD+ peripheral blood B cells expressing the CD27 cell surface antigen carry somatically mutated variable region genes: CD27 as a general marker for somatically mutated (memory) B cells. J Exp Med.

[34] Koenen RR, von Hundelshausen P, Nesmelova IV, Zernecke A, Liehn EA, Sarabi A (2009). Disrupting functional interactions between platelet chemokines inhibits atherosclerosis in hyperlipidemic mice. Nat Med.

[35] Henneken M, Dorner T, Burmester GR, Berek C (2005). Differential expression of chemokine receptors on peripheral blood B cells from patients with rheumatoid arthritis and systemic lupus erythematosus. Arthritis Res Ther..

[36] Nanki T, Takada K, Komano Y, Morio T, Kanegane H, Nakajima A (2009). Chemokine receptor expression and functional effects of chemokines on B cells: implication in the pathogenesis of rheumatoid arthritis. Arthritis Res Ther..

[37] Corcione A, Ferlito F, Gattorno M, Gregorio A, Pistorio A, Gastaldi R (2009). Phenotypic and functional characterization of switch memory B cells from patients with oligoarticular juvenile idiopathic arthritis. Arthritis Res Ther.

[38] Infantino S, Moepps B, Thelen M (2006). Expression and regulation of the orphan receptor RDC1 and its putative ligand in human dendritic and B cells. J Immunol.

[39] Zweemer AJ, Toraskar J, Heitman LH, AP IJ. (2014). Bias in chemokine receptor signalling. Trends Immunol.

[40] Corbisier J, Gales C, Huszagh A, Parmentier M, Springael JY (2015). Biased signaling at chemokine receptors. J Biol Chem.

[41] Shi K, Hayashida K, Kaneko M, Hashimoto J, Tomita T, Lipsky PE (2001). Lymphoid chemokine B cell-attracting chemokine-1 (CXCL13) is expressed in germinal center of ectopic lymphoid follicles within the synovium of chronic arthritis patients. J Immunol.

[42] Legler DF, Loetscher M, Roos RS, Clark-Lewis I, Baggiolini M, Moser B (1998). B cell-attracting chemokine 1, a human CXC chemokine expressed in lymphoid tissues, selectively attracts B lymphocytes via BLR1/CXCR5. J Exp Med.

[43] Baba M, Imai T, Nishimura M, Kakizaki M, Takagi S, Hieshima K (1997). Identification of CCR6, the specific receptor for a novel lymphocyte-directed CC chemokine LARC. J Biol Chem.

[44] Matsui T, Akahoshi T, Namai R, Hashimoto A, Kurihara Y, Rana M (2001). Selective recruitment of CCR6-expressing cells by increased production of MIP-3 alpha in rheumatoid arthritis. Clin Exp Immunol.

[45] Jacobi AM, Dorner T (2010). Current aspects of anti-CD20 therapy in rheumatoid arthritis. Curr Opin Pharmacol.

