# Supplementary material for: Role of CXCL13 and CCL20 in the recruitment of B cells to inflammatory foci in chronic arthritis
Source: Arthritis Res Ther. 2018 Jun 7;20:114. doi: 10.1186/s13075-018-1611-2 (PMC5992813; doi:10.1186/s13075-018-1611-2)
Supplement: Supplementary file 1 — Table S1. Mean fluorescence intensity and percentage of positive cells for each chemokine receptor on PB and SF from arthritis patients and healthy controls. (DOCX 16 kb) [file 13075_2018_1611_MOESM1_ESM.docx]

|  | **Arthritis patients** | | | | | | **Controls** | | | |
| --- | --- | --- | --- | --- | --- | --- | --- | --- | --- | --- |
|  | **MFI** | | | **% positive cells** | | | **MFI** | | **% positive cells** | |
|  | **PB** | **SF** | **p** | **PB** | **SF** | **p** | **PB** | **p*** | **PB** | **p*** |
| **CCR1** | 311±76 | 584±155 | **0.03** | 4±1.06 | 13.5±4.59 | **0.03** | 233±8. | 0.56 | 2.51±0.44 | 0.33 |
| **CCR2** | 688±301 | 1086±251 | **0.02** | 1.08±0.31 | 10.28±3.18 | **0.03** | 446±203 | 0.53 | 3.56±1.12 | 0.13 |
| **CCR3** | 1253±818. | 1443±671 | 0.72 | 6.66±1.97 | 9.54±2.57 | 0.4 | 732±446 | 0.63 | 2.05±0.33 | 0.07 |
| **CCR4** | 1319±483 | 6332±1234 | **0.02** | 12.37±2 | 18.60±5.57 | **0.03** | 798±246 | 0.37 | 9.61±1.84 | 0.34 |
| **CCR5** | 287±112 | 697±400 | 0.23 | 0.28±0.04 | 6.70±2.56 | **0.03** | 60±14 | 0.14 | 0.7 ±0.26 | 0.11 |
| **CCR6** | 12559±112 | 8639±799 | **0.009** | 99.65±0.1 | 93.94±2.27 | **0.04** | 7925±375 | **0.001** | 99.35±0.15 | 0.11 |
| **CCR7** | 1071±667 | 1443±684 | 0.82 | 11.5±2.21 | 7.14±1.73 | 0.06 | 576±71 | 0.51 | 18.56±2.49 | 0.08 |
| **CXCR2** | 89±23 | 120±39 | 0.38 | 0.35±0.11 | 1.27±1.07 | 0.53 | 53±11 | 0.2 | 1.06±0.49 | 0.28 |
| **CXCR4** | 3301±299 | 4725±856 | **0.03** | 87.05±2.52 | 82.18±4.43 | 0.5 | 3526±350 | 0.38 | 89.78±1.37 | 0.25 |
| **CXCR5** | 15429±1607 | 7272±1497 | **0.01** | 98.79±0.27 | 53.5±10.4 | **0.03** | 10372±2991 | 0.11 | 97.86±0.45 | 0.1 |
| **CXCR7** | 425±34 | 209±141 | **0.02** | 5.78±0.86 | 2.94±0.79 | 0.1 | 430±95 | 0.96 | 5.68±0.72 | 0.9 |
| Values are mean±SE of MFI and percentage of positive cells for each chemokine receptor on PB and SF from arthritis patients and healthy controls.  MFI: mean fluorescence intensity; PB: peripheral blood; SF: synovial fluid  p* value using unpaired t-test comparing PB from controls and patients.  p value using paired t-test comparing PB and SF from patients. | | | | | | | | | | |

**Table S1**. Mean fluorescence intensity and percentage of positive cells for each chemokine receptor on PB and SF from arthritis patients and healthy controls.
